# Maximizing the Longevity and Volume Retention of Fat Grafts: Advances in Clinical Practice

**DOI:** 10.7759/cureus.88493

**Published:** 2025-07-22

**Authors:** Pawan Acharya, Cara Mohammed, Arusha Desai, Maria Camila Rojas Gomez, Gopika Sunil, Patricio Xavier Duran S, Sami Kocaekiz, Abrar Ahmed Thottakurichi, Ibzan Janier Gonzalez Munoz, Luis Antonio Chavez-Alvarez, Vivasvat Binny, Manju Rai

**Affiliations:** 1 Surgery, Charing Cross Hospital, London, GBR; 2 Orthopaedic Surgery, Sangre Grande Hospital, Sangre Grande, TTO; 3 Surgery, Armed Forces Medical College, Pune, IND; 4 Faculty of Medicine, Universidad del Rosario, Bogotá, COL; 5 Surgery, Hamdard Institute of Medical Sciences and Research, New Delhi, IND; 6 Internal Medicine, Universidad de Cuenca, Cuenca, ECU; 7 Emergency Medicine, Bakırçay University Çiğli Training and Research Hospital, Izmir, TUR; 8 Faculty of Medicine, Ivane Javakhishvili Tbilisi State University, Tbilisi, GEO; 9 Surgery, Universidad Autonoma de Guadalajara School of Medicine, Guadalajara, MEX; 10 Surgery, Escuela de Medicina y Ciencias de la Salud, Monterrey, MEX; 11 Science, Gretchen Whitney High School, Cerritos, USA; 12 Biotechnology, Shri Venkateshwara University, Gajraula, IND

**Keywords:** adipose-derived stem cells, autologous fat transfer, compact grafting, fat graft survival, platelet-rich plasma, preconditioning, regenerative medicine, scaffold

## Abstract

Autologous fat grafting (AFG) is increasingly utilized in aesthetic and reconstructive surgery owing to its biocompatibility, regenerative properties, and ease of harvest. However, inconsistent graft retention rates pose a persistent clinical challenge. This review explores contemporary advancements aimed at improving fat graft survival and volume retention. Biological enhancements, such as platelet-rich plasma (PRP), stromal vascular fraction (SVF), and adipose-derived stem cells (AdSCs), have demonstrated efficacy in promoting angiogenesis, reducing fibrosis, and improving graft integration. Emerging strategies, including vitamin E (VE) augmentation, compact fat grafting, and soluble molecule preconditioning with agents like deferoxamine (DFX) and vascular endothelial growth factor (VEGF), have shown promise in experimental and clinical models. The synergistic use of PRP and AdSCs yields higher growth factor expression, enhancing tissue viability. Innovations like Botulinum Toxin-A (BoNTA) for muscle immobilization, concentrated de-oiled fat (CDF), and biodegradable scaffolds further contribute to improved outcomes. Site-specific adaptations - for craniofacial, breast, and gluteal regions - demonstrate tailored approaches that enhance regional graft viability.

Despite these advancements, standardization remains a barrier due to methodological heterogeneity in harvesting, processing, and application. Furthermore, while preliminary results from clinical trials are encouraging, long-term data and larger cohorts are required to validate safety and effectiveness. Future research should focus on optimizing stem cell enrichment protocols, biomaterial integration, and understanding molecular pathways that govern graft survival. Collectively, these evolving strategies represent a significant leap forward in maximizing the functional and aesthetic benefits of fat grafting, heralding a new era of regenerative plastic surgery.

## Introduction and background

Autologous fat grafting (AFG) has become a widely adopted technique in both aesthetic and reconstructive surgery due to its ease of harvest, biocompatibility, and regenerative potential [1,2]. Its applications range from facial rejuvenation and breast reconstruction to scar correction and the treatment of radiation-induced fibrosis [3,4]. Adipose tissue is particularly valued not only for volumetric restoration but also for its rich reservoir of adipose-derived stem cells (AdSCs), which contribute to tissue regeneration via paracrine signaling (the process by which cells secrete signaling molecules that act on nearby cells), angiogenesis, and differentiation [5,6].

Despite its advantages, one of the key limitations of fat grafting remains the unpredictable resorption of the grafted tissue. Clinical and experimental studies have reported variable retention rates, ranging from 30% to 70%, necessitating repeated procedures to achieve desired outcomes [7]. This variability stems from multiple factors, including harvesting methods, graft processing techniques, recipient site conditions, and postoperative care [8,9]. These influences affect the viability of transplanted adipocytes and the integration of the graft with the host tissue.

Recent advances in regenerative medicine and surgical technique have focused on enhancing fat graft survival and volume retention. Strategies include cellular enrichment using stromal vascular fraction (SVF) and AdSCs, preconditioning with bioactive molecules, mechanical manipulation, and the use of platelet-rich plasma (PRP) [10-12]. Furthermore, synthetic scaffolds, such as allograft adipose matrices (AAMs), offer alternatives when autologous fat harvesting is not feasible [13].

The continued evolution of fat grafting techniques highlights the need for a comprehensive understanding of the underlying physiology, innovations in clinical application, and standardized methodologies. This narrative review aims to provide a comprehensive overview of current strategies to improve fat graft survival and volume retention, with a focus on biologic enhancements, mechanical techniques, site-specific adaptations, and emerging technologies. By synthesizing recent evidence from preclinical and clinical studies, we highlight both established practices and promising innovations in the field of AFG.

## Review

Stem cells and fat grafting

AdSCs​​​​​​: Biological Foundations

AdSCs represent a groundbreaking frontier in regenerative medicine, particularly within the realm of plastic and reconstructive surgery. These multipotent mesenchymal stem cells, harvested from adipose tissue through minimally invasive liposuction techniques, possess extraordinary regenerative capabilities that extend far beyond traditional tissue replacement. The unique characteristics of AdSCs include their ability to differentiate into multiple cell lineages, including adipocytes, osteoblasts, chondrocytes, and endothelial cells, making them an invaluable resource in surgical reconstruction and aesthetic enhancement.

The molecular sophistication of AdSCs lies in their complex biological mechanisms. These cells secrete an array of critical growth factors, including vascular endothelial growth factor (VEGF), basic fibroblast growth factor (bFGF), and transforming growth factor-beta (TGF-β). These molecular mediators play pivotal roles in promoting angiogenesis, reducing inflammation, and stimulating tissue regeneration. The paracrine signaling capabilities of AdSCs enable them to orchestrate sophisticated healing processes that go beyond simple cellular replacement, effectively transforming the landscape of reconstructive surgical interventions.

Applications in Reconstructive Surgery

Advancements in stem cell-enriched fat grafting have dramatically expanded the potential of traditional fat transfer techniques. The pioneering cell-assisted lipotransfer (CAL) technique, first described by Matsumoto et al. (2006), represents a quantum leap in this field [3]. CAL involves isolating the SVF from harvested adipose tissue, which is rich in stem cells, and then reintroducing these concentrated cells into the primary fat graft [14]. This innovative approach has demonstrated significantly improved graft survival rates, enhanced tissue integration, and more predictable volumetric outcomes compared to conventional fat grafting methods.

The regenerative potential of AdSCs in reconstructive surgery extends across multiple clinical domains. Breast reconstruction following mastectomy has seen particularly promising results, with stem cell-enriched fat grafting offering improved tissue quality, enhanced healing, and more natural aesthetic outcomes [15,16]. Similarly, patients with complex wound healing challenges, including those with radiation-damaged tissues, have benefited from the remarkable regenerative capabilities of AdSCs [17]. The cells' ability to promote angiogenesis and reduce inflammation makes them particularly effective in challenging reconstructive scenarios where traditional techniques often fall short.

Recent research has explored increasingly sophisticated manipulations of AdSCs, including ex vivo expansion, genetic modification, and combined growth factor therapies. These cutting-edge approaches aim to optimize stem cell concentration, enhance proliferative capacities, and develop more targeted delivery mechanisms for specific reconstructive applications. The potential applications continue to expand, with emerging research investigating the use of AdSCs in treating conditions ranging from chronic wounds to complex soft tissue defects.

Clinical Trials With Adipose-Derived Mesenchymal Stem Cells (AdMSCs)

Several clinical trials have been conducted in the recent past investigating AdMSC therapy for plastic surgery. Fernández et al. (2018) conducted a triple-blind, placebo-controlled study to evaluate the safety and feasibility of autologous AdMSC infusion in patients with secondary progressive multiple sclerosis (SPMS) [11]. In this study, 34 patients underwent liposuction to harvest excess fat, which was subsequently expanded for AdMSC collection. Patients were administered either a low dose (1 × 10^6^ cells/kg) or a high dose (4 × 10^6^ cells/kg) AdMSC product, with a placebo control [11]. The follow-up period extended over 12 months, during which adverse events, laboratory parameters, vital signs, and spirometry were monitored. One serious adverse event, a urinary infection, was observed in the treatment arms; however, it was deemed unrelated to the study treatment. Overall, the study concluded that the infusion of autologous AdMSCs is safe and feasible in this patient population.

Freitag et al. (2019) conducted a randomized controlled trial to evaluate the safety and efficacy of autologous AdMSC therapy in individuals with symptomatic knee osteoarthritis [18]. Healthy human adipose tissue was harvested to derive AdMSCs, and 30 participants were randomized to receive either a single injection (100 × 10^6^ AdMSCs) or two injections (100 × 10^6^ AdMSCs). The study followed the participants over a period of six months, monitoring safety, pain levels, and functional changes. No serious adverse events were reported in either treatment group. At the 12-month follow-up, both groups demonstrated clinically significant improvements in pain and functional outcomes. The study concluded that AdMSC therapy is a safe and effective treatment option for knee osteoarthritis, with the potential to slow down or even prevent the progression of the disease.

Gentile et al. (2020) presented an observational case-series study assessing the safety and efficacy of fat grafting enhanced with adipose-derived stem cells (FG-e-ASCs) for breast augmentation in patients affected by breast hypoplasia [19]. The donor sites included the abdomen, flanks, and inner knees. A total of 46 patients were treated with fat grafting procedures, with an average injection volume of 180 mL (ranging from 80 to 280 mL). The follow-up period lasted 48 weeks, during which clinical evaluations, magnetic resonance imaging (MRI), photographic assessments of soft tissue, mammography, and ultrasound were performed. Patients treated with FG-e-ASCs demonstrated a 58% maintenance of contour restoration, and 67.4% of the treated patients experienced a restoration of the breast contour. The study concluded that the use of FG-e-ASCs was both safe and effective in this case series.

de Celis-Ruiz et al. (2022) conducted a randomized, double-blind, placebo-controlled, single-center pilot clinical trial to investigate the safety of allogeneic AdMSCs in ischemic stroke patients [20]. AdMSCs were harvested from a healthy donor and expanded in a laboratory setting. Fifteen patients participated in the study, with four receiving AdMSCs and nine receiving a placebo (saline solution at 1 mL/kg of body weight). Each patient in the AdMSC group received 10^6^ cells/kg of body weight. The follow-up extended over 24 months, during which the safety profile was assessed through adverse events, neurologic and systemic complications, and tumor monitoring. The total number of adverse events and systemic or neurologic complications was similar between the treatment and placebo groups, with no injection-related adverse events or tumor development observed. At 24 months, patients in the AdMSC group exhibited a nonsignificantly lower median National Institutes of Health Stroke Scale (NIHSS) score. The study concluded that intravenous administration of AdMSCs within the first two weeks following ischemic stroke is safe over a 24-month follow-up period.

The results of these studies support that infusion of autologous AdMSCs is safe and feasible in patients with SPMS. Clinical studies show that FG-e-ASCs are both safe and effective [6]. More studies suggest that the administration of AdMSCs directly to the blood after ischemic stroke is safe when done in the first two weeks, and is safe for 24 months [19,21].

Site-specific strategies for enhancing fat graft survival

Craniofacial Region

The face is among the most common sites for AFG, particularly for contour correction, volume loss due to aging, and post-traumatic reconstruction. Strategies such as SVF enrichment, PRP pre-treatment, and microdroplet injection techniques have been shown to significantly improve graft retention in this region [8,10,22]. In a multicenter randomized clinical trial (RCT), SVF-enriched grafts achieved a 74.5% survival rate at six weeks and 71.3% at 24 weeks, with particularly enhanced retention in the forehead (12.82% higher) and cheeks, compared to controls (p < 0.035) [10].

Adjuncts such as vitamin E (VE) have demonstrated reductions in oxidative stress and improvements in angiogenesis, further supporting graft survival in irradiated or fibrotic facial tissues [23]. The application of facial-derived stem cells (FdSCs) has also shown promise in the craniofacial region, due to their superior angiogenic potential over AdSCs [11].

Breast and Chest Wall

In reconstructive breast surgery, particularly after mastectomy or radiation therapy, improving fat graft survival is critical. Integration of SVF, VEGF-rich preconditioning, and scaffold-based support, such as AAM, has shown enhanced retention and remodeling outcomes [10,18,24]. PRP and CAL are increasingly used to counteract the hostile, fibrotic recipient environment [22].

Gluteal and Body Contouring

Gluteal augmentation with fat is volume-intensive and carries a higher risk of fat necrosis if large volumes are poorly vascularized. Techniques like multiplanar fat deposition, subcutaneous-only injection protocols, and nutrient-rich pre-treatment of fat (e.g., PRP or antioxidants) have been employed to improve safety and retention [18,23].

Innovations in fat graft longevity and survival

Fat grafting, while an attractive and seemingly straightforward procedure, presents significant clinical challenges - most notably tissue necrosis and resorption - with studies reporting graft loss rates of 40%-60% [25]. The success of the procedure, as with any surgical intervention, hinges on three critical factors: the specific technique employed, the surgeon's expertise and technical proficiency, and the precise execution of the procedure.

The method of fat harvesting significantly influences graft viability. Research has demonstrated that laser-assisted liposuction results in markedly reduced tissue viability compared to ultrasound- and suction-assisted approaches [26]. Among various processing techniques, the cotton gauze rolling method has emerged as superior to both filtration and centrifugation methods in preserving graft quality [27].

PRP and CAL Techniques

Recent advances in the field have introduced promising enhancement techniques, particularly PRP and CAL, which have substantially improved graft survival rates. PRP has proven especially effective in enhancing volumetric retention while remaining both accessible and cost-effective [26]. The mechanism involves activated platelets releasing growth factors that facilitate tissue repair, with peak activity in the first hour and sustained effects for approximately seven days [28]. Key growth factors, including platelet-derived growth factor-AB (PDGF-AB), VEGF, epidermal growth factor (EGF), and TGF-β1, stimulate proliferation of human AdSCs and dermal fibroblasts [29]. These factors significantly enhance angiogenesis, cell differentiation, and extracellular matrix formation, ultimately improving cellular resistance to hypoxic stress.

Recent advances have highlighted the remarkable potential of AdSCs when implemented through CAL techniques [3]. Clinical observations demonstrate that CAL-enhanced grafts exhibit a 35% higher survival rate compared to traditional non-CAL (NCAL) procedures [3]. This enhanced viability stems from the unique capacity of AdSCs to undergo cellular differentiation, effectively replacing adipose cells lost to hypoxic or physical stress.

A particularly noteworthy aspect of AdSCs is their adaptive response to hypoxic conditions, wherein they demonstrate increased production of critical growth factors, specifically VEGF and bFGF. This upregulation not only enhances cellular proliferation but also significantly improves tissue survivability and wound healing capabilities [30,31]. Even more compelling are the synergistic effects observed when combining PRP with AdSCs. This combination therapy yields substantially higher growth factor levels compared to either treatment alone, resulting in superior outcomes in terms of both graft volume preservation and weight maintenance [32].

Novel Enhancement Approaches

Recent innovations in fat grafting techniques have introduced novel approaches to enhance graft survival. Among these, Baek et al. (2012) pioneered the use of Botulinum Toxin-A (BoNTA) for fat graft immobilization [33]. The enhanced graft survival observed with this method is attributed to BoNTA's muscle-paralyzing effect, which reduces underlying tissue contractions and preserves graft viability. Additionally, BoNTA has been shown to promote angiogenesis through both the mTOR/HIF1α/VEGF and NF-κB/HIF1α/VEGF pathways [34]. While initial results are promising, further clinical trials are necessary to establish conclusive evidence regarding safety, optimal dosing protocols, and long-term outcomes, before this approach can transition from experimental to standard clinical practice [34].

In the realm of biomaterial science, biodegradable and biocompatible polymers, particularly hyaluronic acid and polyglycolic acid, are emerging as effective functional scaffolds. These materials promote adipose tissue regeneration while gradually degrading, facilitating enhanced neovascularization and protecting autologous fat from in vivo degeneration [35]. Notably, alginate-fat scaffolds have demonstrated superior adipogenesis properties and reduced immunological responses compared to conventional fat injection or alginate gels alone, as evidenced by four-week post-transplantation studies [35]. However, while these bioactive scaffolds show promise, additional research is required to validate their clinical efficacy and safety for routine use [36].

Further advancing the field, research by Hu et al. (2018) has demonstrated the effectiveness of concentrated growth factors (CGFs) in promoting adipose tissue regeneration [37]. In studies using nude mice, CGFs - which contain CD34 stem cells within a small plasma volume - exhibited enhanced tissue regeneration and stability, characterized by increased vascularity and reduced fibrosis [37].

Recent evidence-based strategies to improve fat graft survival

PRP Enhancement

A recent meta-analysis of 15 trials involving 1,215 patients found that PRP-supplemented grafts significantly increased fat retention (effect size = 0.34, p < 0.00001), reduced recovery times, and elevated patient satisfaction compared to fat grafts alone [38]. The study reported that PRP facilitates early vascularization by releasing growth factors that reduce ischemic damage and enhance adipocyte survival. Another randomized animal trial reported that PRP preconditioning improved vascularization and reduced fibrosis within grafts, suggesting superior tissue integration [39].

Soluble Molecule Preconditioning

Compounds like deferoxamine (DFX), melatonin, and VEGF enhance adipogenesis and vascularization. DFX stabilizes hypoxia-inducible factors, promoting VEGF expression and vascular ingrowth [40]. Preconditioning irradiated recipient sites with DFX iron overload (DFO) has been reported to induce neovascularization and significantly increase fat graft retention in rat models [41]. A study found that using VEGF, either as preconditioning or at the time of fat grafting, significantly improved fat graft viability and adipocyte preservation in rats compared to controls. The results suggest that VEGF enhances revascularization, making it an effective strategy to increase graft survival [42]. These strategies remain promising, though standardization of dosing protocols is needed.

SVF and Stem Cell Approaches

SVF-enriched grafts continue to demonstrate superior retention in both clinical and preclinical studies. A multicenter RCT demonstrated that SVF enrichment significantly improved fat graft survival rates in facial fat transfer, with MRI-confirmed retention rates of 74.5% at six weeks and 71.3% at 24 weeks, compared to lower rates in the control group [43]. The procedure was deemed safe, with no postoperative complications or bacterial contamination observed, and showed superior aesthetic outcomes in surgeon evaluations. Chen et al. (2025) demonstrated that FdSCs exhibit significantly greater angiogenic potential than AdSCs, with higher expression of HMOX1, HIF-1α, and VEGFa [44]. Both in vitro and in vivo experiments confirmed that FdSCs enhance vascularization and fat graft retention, suggesting their promising role in improving outcomes in soft tissue regeneration.

Another clinical observation found that the synergistic use of PRP and ADSCs resulted in significantly higher levels of VEGF, improving angiogenesis and reducing fat necrosis rates by more than 40%, compared to non-enriched grafts [45].

Compact Fat Grafting

Kim et al. (2021) introduced compact fat grafting, which reduced adipocyte size using MLN4924, a NEDD8-activating enzyme inhibitor [46]. In a C57BL/6J murine model, the high-dose MLN4924 group showed a 39% reduction in adipocyte size and significantly improved fat retention at four and eight weeks. Histological analysis confirmed lower hypoxia levels and a favorable M2 macrophage response - conditions optimal for graft survival.

Mechanical Stress and Postoperative Considerations

Chun et al. (2022) demonstrated that applying mechanical stress to adipose tissue significantly enhances the proliferation capacity of AdSCs without affecting their differentiation potential [47]. When used in CAL, AdSCs derived from mechanically stimulated fat improved graft engraftment, suggesting a clinically feasible method to enhance CAL efficacy. Postoperative care, including avoiding pressure, trauma, and risk factors like smoking, is essential to improving retention [48].

Concentrated De-oiled Fat (CDF)

Yang et al. (2024) developed a CDF technique, which involved flocculation and centrifugation to remove oil - a known inflammatory trigger [49]. In murine models, both low- and high-volume CDF grafts retained more volume and demonstrated reduced fibrosis and inflammation after eight weeks, with up to 40% improved retention compared to Coleman fat.

Researchers developed a method to obtain ultra-condensed fat (UCF) by de-oiling intact fat components, aiming to improve fat graft retention [50]. In a nude mouse model over 90 days, UCF demonstrated improved structural integrity and vascularization compared to standard fat grafts. A retrospective study evaluated the use of condensed low-density fat combined with high-density fat in breast reconstruction [51]. Patients receiving the combined grafting technique showed improved fat retention rates and reduced complications, like oil cyst formation, compared to conventional methods.

VE Augmentation

Abbas et al. (2022) found that locally administered VE significantly improved fat graft volume retention and reduced radiation-induced fibrosis in irradiated mice, as early as one week post-grafting [52]. VE-treated grafts showed enhanced dermal thickness, reduced oxidative stress, increased angiogenesis, and favorable cytokine profiles compared to untreated and pentoxifylline-treated groups. Cinar et al. (2024) investigated the effects of various antioxidants, including VE, on fat graft survival in rats [53]. The VE group showed a significant increase in total antioxidant capacity and improved graft volume retention compared to controls.

Synthetic Scaffolds and Biologics

New-generation biomaterials like alginate-fat scaffolds and AAM (e.g., Renuva®) have achieved promising results. A multicenter study demonstrated 75% volume retention at six months using AAM, with high patient satisfaction in facial, hand, and body treatments [54]. These matrices support angiogenesis and integrate without provoking immune responses, offering practical alternatives when autologous fat is unavailable.

A proof-of-concept study demonstrated that 3D-printed bioresorbable scaffolds can enhance the structural integrity and vascularization of fat grafts [55]. These scaffolds mimic the extracellular matrix, providing a supportive environment that promotes adipocyte survival and integration. While specific percentages vary, the study reported improved volume retention compared to fat grafting without scaffolds. Nanofat technology, combining emulsified fat, AdSCs, and growth factors, has emerged as another effective approach for superficial tissue regeneration, particularly in scar management and skin rejuvenation [56]. Its effectiveness in scar treatment is attributed to its unique cellular composition and its ability to enhance vascularity while providing anti-apoptotic and anti-inflammatory benefits [57].

Complications and safety considerations

AFG has emerged as a prominent technique in both aesthetic and reconstructive surgery, distinguished by its unique advantages as a soft tissue filler. These benefits include readily available donor tissue, straightforward harvesting procedures, cost-effectiveness, minimal donor site complications, and low immunological response [58].

Comprehensive preoperative assessment forms the primary component of successful outcomes. This crucial evaluation identifies potential risk factors, such as thrombotic history, immunocompromised status, and coagulation disorders, enabling optimal patient selection and risk stratification [59]. Thorough preoperative counseling regarding procedure-specific benefits and risks constitutes another vital component. This communication not only enhances patient satisfaction but also minimizes potential post-surgical conflicts and disappointment [60].

The technical aspects of harvesting, processing, and injection significantly influence postoperative outcomes. Small-caliber cannulas and precise injection techniques have demonstrated a reduced risk of neurovascular injury. Subcutaneous plane injection is strongly preferred to minimize fat embolism risk, a potentially lethal complication. Graft volume selection requires careful consideration, as larger volumes correlate with increased fat necrosis and reduced long-term survival rates. Additionally, minimally traumatic techniques are paramount in preserving adipocyte viability, optimizing graft integration, and minimizing fibrosis and contour irregularities [60,61]. Surgical expertise in managing both intraoperative and postoperative complications remains essential. Vigilant postoperative monitoring enables early detection and intervention for potential complications [62].

Advanced technologies, particularly intraoperative ultrasound guidance, are gaining traction for precise graft placement and vital structure preservation. While these innovations show promise for reducing complications and enhancing placement accuracy, further research is needed to establish their clinical efficacy, safety profile, and cost-effectiveness [62,63].

Challenges and future research

Despite its widespread adoption, fat grafting faces significant clinical challenges, particularly regarding graft retention and predictability. Additionally, despite encouraging short- to mid-term results, many current studies lack extended follow-up periods. This limits our understanding of the long-term sustainability and safety of these graft enhancement techniques. Post-procedure resorption remains a major concern, with studies reporting volume loss ranging from 20% to 80%, often necessitating multiple interventions to achieve desired outcomes [64]. While AdSCs possess regenerative properties that enhance graft survival through angiogenesis and tissue integration, their efficacy in fat grafting remains controversial. This uncertainty stems from methodological variations in injection techniques, graft volumes, AdSC concentrations, and recipient sites, making standardization of outcomes challenging [65]. Additionally, individual patient factors, including metabolism and local tissue environment, significantly influence graft success and long-term volume retention [66]. Although AdSCs demonstrate potential benefits in reconstructive procedures and sclerotic conditions, their effectiveness continues to generate debate within the scientific community [67].

Future research directions may focus on modifying AdSCs to produce optimal growth factor profiles, potentially enhancing graft viability and reducing the need for repeated procedures. While AdSCs demonstrate improved angiogenesis and tissue integration, the underlying mechanisms - whether through paracrine effects or cellular differentiation - remain unclear. Increased VEGF expression appears to enhance blood flow through angiogenesis or vasodilation and accelerate wound healing, though further investigation of molecular mechanisms is needed [68]. Despite promising preclinical results from combining AdSCs with growth factors and biological scaffolds, additional clinical trials are essential to validate their safety and efficacy [69].

Advancing this field requires a coordinated global effort combining multidisciplinary expertise to establish reproducible protocols for AdSC expansion, scaffold preparation, and clinical application. The integration of existing AdSC research with emerging technologies, particularly 3D printing, may provide more standardized and reliable approaches for both aesthetic and reconstructive surgical applications.

## Conclusions

Advancements in fat grafting in aesthetic and reconstructive surgery have equipped clinicians with a highly effective tool for customized soft tissue restoration. Improved methods of harvesting, processing, and grafting fat, along with the integration of regenerative technologies such as stem cells and their derivatives, have addressed longstanding issues of graft retention variability and outcome inconsistency. As research advances, future developments may include optimized protocols for stem cell-enriched grafts, sophisticated methods for graft survival assessment, and improved integration with 3D imaging. In totality, these advances further cement fat grafting as a foundation of contemporary surgical practice, significantly enhancing patient outcomes and experience.
